# Induction of Fetal Hemoglobin by Introducing Natural Hereditary Persistence of Fetal Hemoglobin Mutations in the γ-Globin Gene Promoters for Genome Editing Therapies for β-Thalassemia

**DOI:** 10.3389/fgene.2022.881937

**Published:** 2022-05-17

**Authors:** Dian Lu, Zhiliang Xu, Zhiyong Peng, Yinghong Yang, Bing Song, Zeyu Xiong, Zhirui Ma, Hongmei Guan, Bangzhu Chen, Yukio Nakamura, Juan Zeng, Nengqing Liu, Xiaofang Sun, Diyu Chen

**Affiliations:** ^1^ Department of Obstetrics and Gynecology, Center for Reproductive Medicine/Department of Fetal Medicine and Prenatal Diagnosis/BioResource Research Center, Key Laboratory for Major Obstetric Diseases of Guangdong Province, The Third Affiliated Hospital of Guangzhou Medical University, Guangzhou, China; ^2^ Nanfang-Chunfu Children’s Institute of Hematology, Taixin Hospital, Dongguan, China; ^3^ Cell Engineering Division, RIKEN BioResource Center, Tsukuba, Japan

**Keywords:** fetal hemoglobin (HbF), hereditary persistence of fetal hemoglobin (HPFH), thalassemia, genome editing, adeno-associated virus (AAV)

## Abstract

Reactivation of γ-globin expression is a promising therapeutic approach for β-hemoglobinopathies. Here, we propose a novel Cas9/AAV6-mediated genome editing strategy for the treatment of β-thalassemia: Natural HPFH mutations −113A > G, −114C > T, −117G>A, −175T > C, −195C > G, and −198T > C were introduced by homologous recombination following disruption of BCL11A binding sites in *HBG1/HBG2* promoters. Precise on-target editing and significantly increased γ-globin expression during erythroid differentiation were observed in both HUDEP-2 cells and primary HSPCs from β-thalassemia major patients. Moreover, edited HSPCs maintained the capacity for long-term hematopoietic reconstitution in B-NDG hTHPO mice. This study provides evidence of the effectiveness of introducing naturally occurring HPFH mutations as a genetic therapy for β-thalassemia.

## Introduction

β-thalassemia is a common monogenic disorder caused by *HBB* gene mutations that alter quantity or quality of the β-polypeptide of adult hemoglobin (HbA, α2β2), with an annual incidence of one in 100,000 worldwide (Berntorp and Shapiro, 2012). The average life expectancy of patients suffering from severe hemolytic anemia is only approximately 30 years even when they are able to receive intensive medical care, mainly involving regular transfusion and iron chelation therapy. Allogeneic stem cell transplantation, the only curative therapy for β-thalassemia, is limited by donor availability and transplant-associated medical complications (Bernaudin et al., 2017). Hence, gene-editing-based autologous hematopoietic stem therapy are being explored.

Hereditary persistence of fetal hemoglobin (HPFH) is a benign condition in which genetic variations attenuate γ-to β-globin switching, causing elevated and persistent production of fetal hemoglobin (HbF, α2γ2). Co-inheritance of HPFH with β-thalassemia ameliorates their clinical severity (Chen et al., 2017). Single-nucleotide polymorphisms (SNPs) associated with HPFH within the proximal fetal γ-globin gene promoters have been identified at positions −113, −114, −117, −175, −195, −196, −197, −198, −201, and −202 upstream of *HBG1/HBG2* transcription start sites (Wienert et al., 2018). Among these, −113A > G, −175T > C, and −198T > C have been reported to create *de novo* binding sites for the potent activators GATA1 (Martyn et al., 2019), TAL1 (Wienert et al., 2015), and KLF1 (Wienert et al., 2017) respectively, while other mutations disrupt the binding sites for HbF-repressors BCL11A (−114C > T, −117G>A) or ZBTB7A (−195C > G, −196C > T, −197C > T, −201C > T, −202C > T) (Wienert et al., 2018). Previous studies have shown that CRISPR/Cas9-mediated disruption of the BCL11A or ZBTB7A binding site in the *HBG1/HBG2* promoters with high on-target frequency leads to significant re-activation of γ-globin expression (Métais et al., 2019; Weber et al., 2020). Therefore, introducing HPFH mutations in the *HBG1/HBG2* promoters can be used as a promising strategy for the treatment of β-thalassemia and sickle cell disease (SCD). Here, we mimicked natural HPFH mutations −113A > G, −114C > T, −117G>A, −175T > C, −195C > G, and −198T > C by delivery of an AAV6 homology repair template, followed by immediate electroporation of Cas9-ribonucleoprotein (RNP) complexes specific to the BCL11A binding site and then tested this system in both HUDEP-2 and HSPCs from β-thalassemia major patients. Our studies provide a novel and feasible genome-editing strategy for treating β-thalassemia.

## Materials and Methods

Circulating G-CSF-mobilized human HSPCs and cord blood HSPCs were enriched by immunomagnetic bead selection (Miltenyi Biotec, Bergisch Gladbach, Germany). All samples were processed after approval by the local medical ethics committee. HSPCs and HUDEP-2 were edited with RNP using a Neon Transfection System (Thermo Fisher Scientific, Carlsbad, CA, United States). Approximately 5×10^5^ CD34^+^ cells were transplanted into 8-week-old B-NDG hTHPO mice 7 days after genome editing. The mouse bone marrow was analyzed for human cell chimerism and multi-lineage differentiation 16 weeks after transplantation. Further details on genome editing, genotype analysis, colony formation assay, Giemsa stain, flow cytometry analysis and quantitative reverse-transcription PCR (qPCR) are provided in the Supplementary Material.

## Results

### Efficient Editing at the *HBG* Promoters Induces HbF Expression in Adult HUDEP-2

**FIGURE 1:**
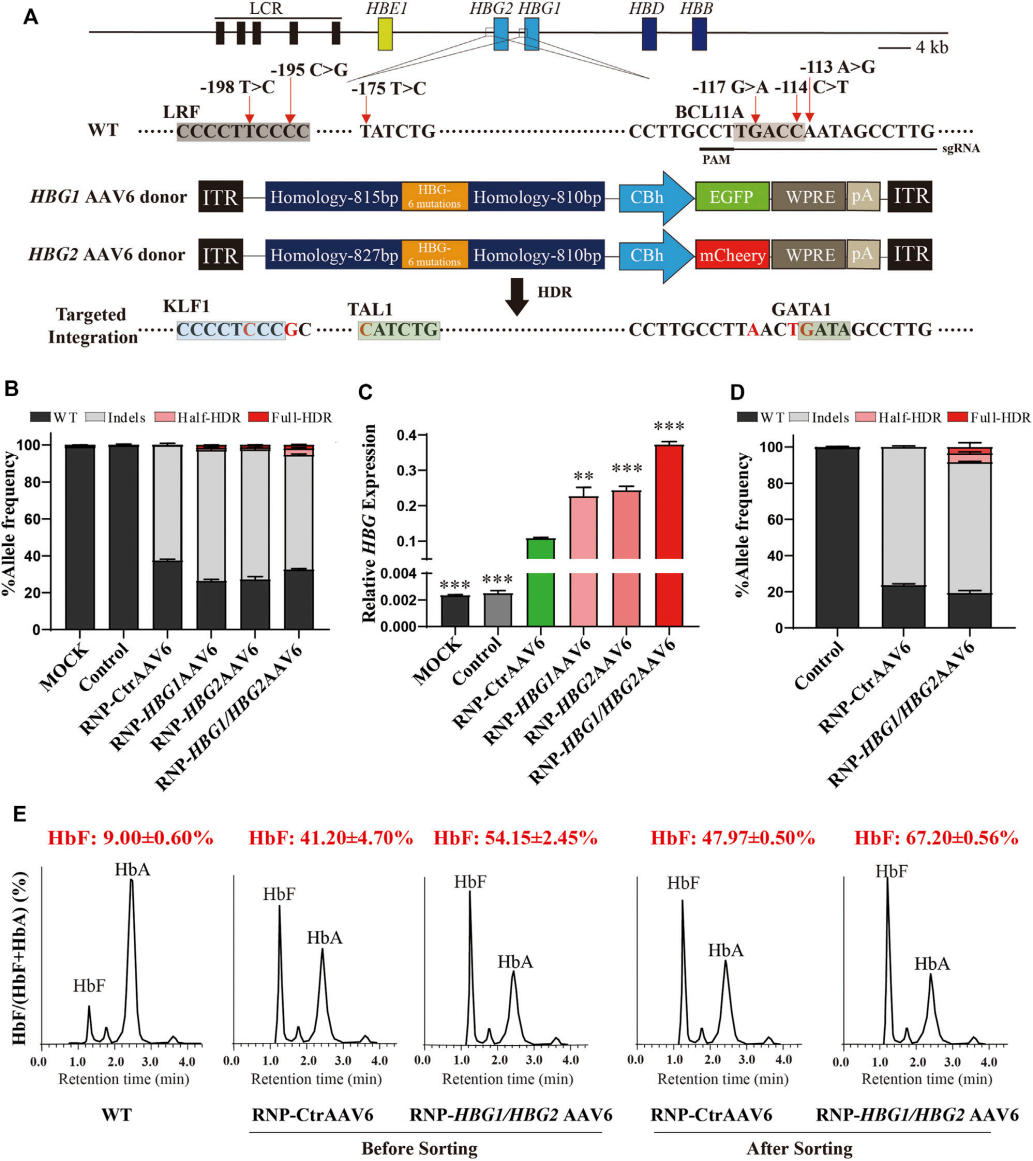
Genome editing of *HBG1/HBG2* promoters reactivate γ-globin expression in HUDEP-2 cells. **(A)** Schematic view of targeted genome editing at the *HBG1/HBG2* promoters using CRISPR/Cas9 and AAV6. Guide RNA spacer sequence and protospacer adjacent motif (PAM) are underlined. Site-specific HPFH mutations are indicated by a red arrow. A DSB stimulates HDR using the AAV6 homologous donor as a repair template. Legend: Orange boxes, *HBG1/HBG2* promoters with six specific HPFH mutations; deep blue boxes, homology arms; green or red boxes, GFP or mCherry expression cassette. **(B)** InDels and HDR frequencies measured by NGS after RNP electroporation and AAV6 transfection in HUDEP-2 cells. **(C)** γ-globin/β-like globin mRNA expression determined by RT-qPCR. **(D)** GFP-positive population were sorted from the group edited by RNP combined with *HBG1/HBG2* AAV6, or from the group edited by RNP combined with CtrAAV6. NGS analysis was performed to identify InDels and determine HDR frequencies. **(E)** The percentage of HbF in HUDEP-2 cells were determined by HPLC on day 8 of differentiation before or after GFP^+^ sorting. The data in Figures 1B,D are presented as the mean ± SD, *n* = 2. In other figures, the data are presented as the mean ± SD, *n* = 3. Student’s *t*-tests were performed to analyse the data. *, *p* < 0.05; **, *p* < 0.01; and ***, *p* < 0.001 vs*.* RNP-CtrAAV6.

We used the chemically modified synthetic sgRNA, which was reported to disrupt the BCL11A binding in *HBG1/HBG2* promoters with high on-target frequency (Métais et al., 2019). We designed two single-stranded AAV6 homology repair vectors (*HBG1* AAV6 and *HBG2* AAV6) to mimic HPFH mutations −113A > G, −114C > T, −117G>A, −175T > C, −195C > G, and−198T > C using approximately 800-bp homologous arms flanking the Cas9 RNP-induced cut site. In parallel, CtrAAV6 lacking homologous recombination elements was introduced as control. *HBG*1 AAV6 and CtrAAV6 were inserted with a GFP expression cassette and *HBG*2 AAV6 with mCherry to validate the effectiveness of transfection (Figure 1A).

To explore the possible effect of AAV6 donor on homologous recombination efficiency, we edited the immortalized erythroid precursor HUDEP-2 cells by adding the AAV6 vector immediately following electroporation of Cas9-RNP complexes. Genome editing results indicated that 62.75 ± 0.93% of InDels were edited by RNP with CtrAAV6, while RNP-*HBG1* AAV6, RNP-*HBG2* AAV6, and RNP-*HBG1/HBG2* AAV6 had similar InDels efficiency, along with a certain degree of homologous recombination efficiency. Importantly, the combination of *HBG*1 AAV6 and *HBG*2 AAV6 tends to increase HDR efficiency, thus leading to a higher mRNA ratio of γ-globin to β-like globin (Figures 1B,C) compared to RNP-CtrAAV6. Unexpectedly, two forms of homologous recombination were successfully introduced in cells edited by RNP with *HBG1* and/or *HBG2* AAV6. One is a complete recombination of six mutations, while the other one is an incomplete homologous recombination only at positions −113, −114, and −117 (Figure 1B), which might be caused by a mismatch of *HBG2* AAV6 donor to *HBG1* in the process of homologous recombination or vice versa. To further explore the editing effect, we isolated the GFP-positive population from the groups edited with *HBG1/HBG2* AAV6 or CtrAAV6. Genome editing results indicated a higher frequency of InDels (62.36 ± 0.43% unsorted, 72.39 ± 0.35% sorted) and total HDR efficiency (5.24 ± 0.21% unsorted, 8.28 ± 1.61% sorted) after GFP-sorting in RNP-*HBG1/HBG2* AAV6 group (Figures 1B,D). Ion-exchange high-performance liquid chromatography (HPLC) assays showed that cells treated with integration of HPFH mutations further increased HbF expression than those edited only with RNP complexes, and this effect was augmented after GFP-sorting (Figure 1E). These results demonstrated the effectiveness of introducing specific HPFH-associated mutations in *HBG1/HBG2* promoters by our genome-editing system.

### Genome Editing of HSPCs and Repopulation of Edited HSPCs *in vivo*

**FIGURE 2:**
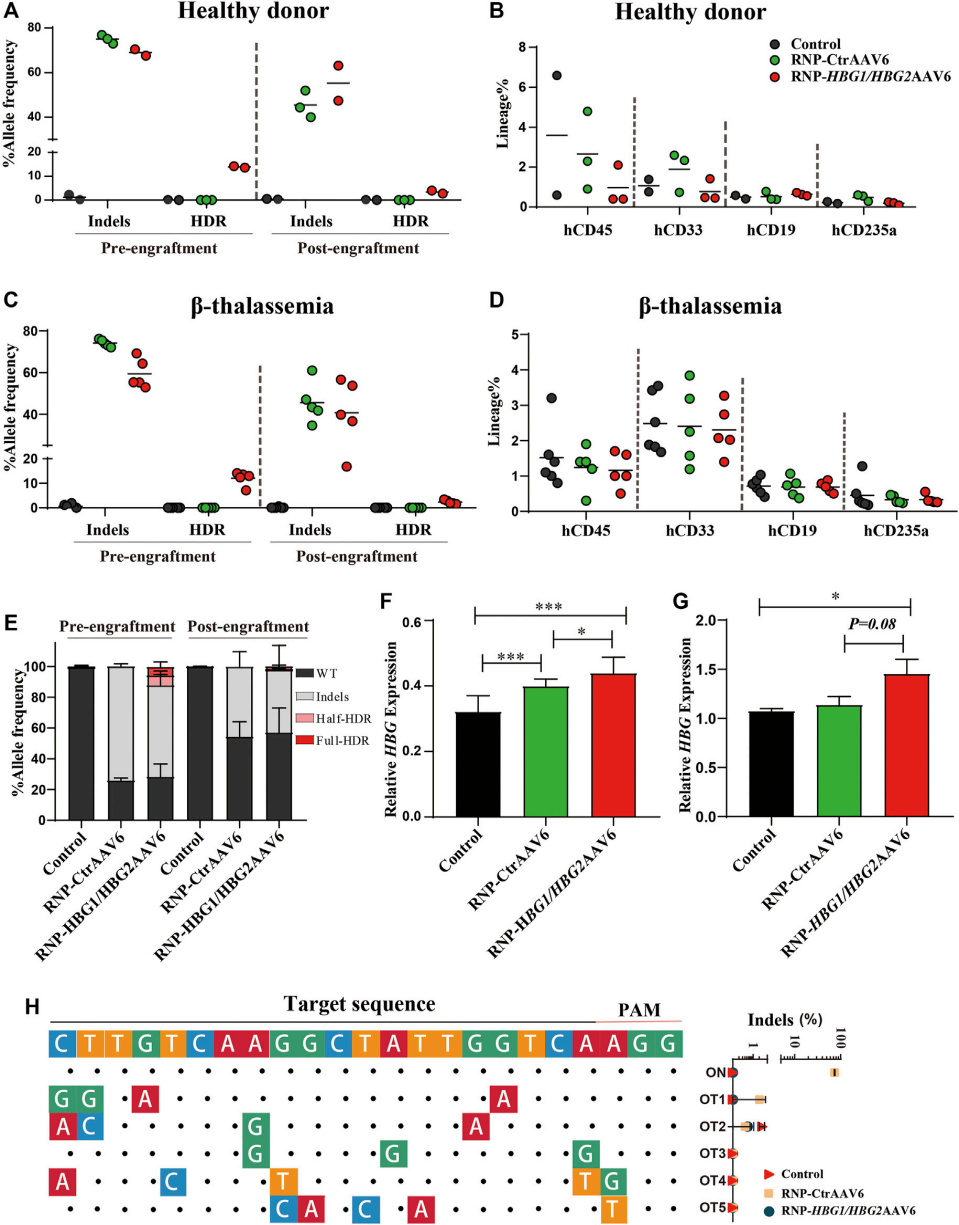
Gene editing of HSPCs induces γ-globin expression and xenotransplantation of gene-edited HSPCs into NBSGW mice. Donor-cell progeny were analyzed in recipient bone marrow (BM) 16 weeks after transplantation. **(A,C)** Different genotype frequencies in the HSCs of one healthy donor **(A)** and two thalassemia donors **(C)** were determined by NGS before or after transplantation. **(B,D)** Flow cytometry analysis of human CD45^+^, CD33^+^, CD19^+^, and CD235a^+^ cell proportions in mouse bone marrow, which were grafted with HSCs from one healthy donor **(B)** and two thalassemia donors **(D)** at 16 weeks after transplantation. **(E)** Percentage of different genotypes frequencies in HSPCs of two thalassemia donors measured by NGS before or after transplantation. **(F)** Following differentiation of HSPCs into erythrocytes at day 14 *in vitro*, γ-globin mRNA expression determined by RT-qPCR in edited or non-edited cells of thalassemia donor. **(G)** γ-globin mRNA expression in hCD235a^+^ erythroblasts isolated from recipient bone marrow of thalassemia donor. **(H)** The left panel shows the predicted top-ranked off-target sites for RNP. The on-target sites are shown at the top. The matched nucleotides of the candidate off-target sequence aligned to the on-target sequence are indicated by dots, and the unmatched are shown as colored nucleotides. The right panel shows the InDels frequencies. The predicted off-target sites detected by NGS with 15,000 reads and 0.01% threshold. Figures 2A–D: Dots represent biologically independent experiments. Figures 2E–G: Data are presented as the mean ± SD, *n* ≥ 3. Student’s *t*-tests were performed to analyse the data. *, *p < 0.05*; **, *p < 0.01*; and ***, *p < 0.001.*

To test the effect in a clinically relevant model, we tested this gene editing strategy in CD34^+^ HSPCs derived from cord blood of a healthy individual donor. The results show that the editing frequency of InDels was 75.02 ± 1.92% in the RNP-CtrAAV group, while the editing efficiency of InDels and HDR was 69.02 ± 2.02% and 13.89 ± 0.41% in the RNP-*HBG1/HBG2*AAV6 group, respectively (Figure 2A). However, in order for gene modification to be reflected *in vivo*, edited stem cells must be engrafted, repopulate and persist within recipient (Notta et al., 2011). To assess the capacity of repopulation and formation of differentiated blood cells, edited and non-edited HSPCs were injected into B-NDG hTHPO immunodeficient mice. After xenotransplantation for 16 weeks, edited and non-edited HSPCs similarly populated the bone marrow and showed comparable fractions of human T cells (CD33^+^), B cells (CD19^+^), and erythroid cells (CD235a^+^) (Figure 2B). In addition, the editing frequencies of InDels and HDR were reduced in the RNP-*HBG1/HBG*2AAV6 group at week 16 post-transplantation (InDels was 55.30 ± 11.17%, HDR was 3.38 ± 0.90%), similar to what has been reported previously (Métais et al., 2019). It may reflect a reduced engraftment capacity of edited cells. It is unclear if this is due to gene editing alone or a combination of the loss of stemness *in vitro* culture of the transplanted cells. We further investigated this gene-editing strategy in CD34^+^ HSPCs derived from mobilized peripheral blood of two severe β-thalassemia patients, whose genotypes are IVS-II-654/CD17 and CD41-42/CD41-42, respectively. In South China, the common mutations are CD41/42, CD17 (A>T), - 28 (A>G) and IVS-II-654 (C>T) which account for 86.0% of the cases studied (Li, 2017). Genome editing and transplantation experiments suggested that editing frequency and repopulation capacities in β-thalassemia HSCs were similar to the healthy donor before and after transplantation (Figures 2C,D). Additionally, there were no significant differences in total editing efficiency between RNP-CtrAAV and RNP-*HBG1/HBG2*AAV6 (Figure 2E). However, γ-globin mRNA expression was more induced by RNP-*HBG1/HBG2*AAV6 than RNP-CtrAAV *in vitro-* and *in vivo-*derived erythroid cells (Figures 2F,G). We detected top-rank potential off-target candidate sites by next-generation sequencing (NGS) of PCR products generated from edited HSPCs of β-thalassemia donor, wherein no editing-associated mutations were detected at these sites (Figure 2H). These results indicate the safety of the RNP-*HBG1/HBG2*AAV6 group and the stronger effectiveness compared to the RNP group.

### Introducing Hereditary Persistence of Fetal Hemoglobin Mutations in the *HBG1/HBG2* Promoters has no Significant Impact on the Erythroid Cell Differentiation

**FIGURE 3:**
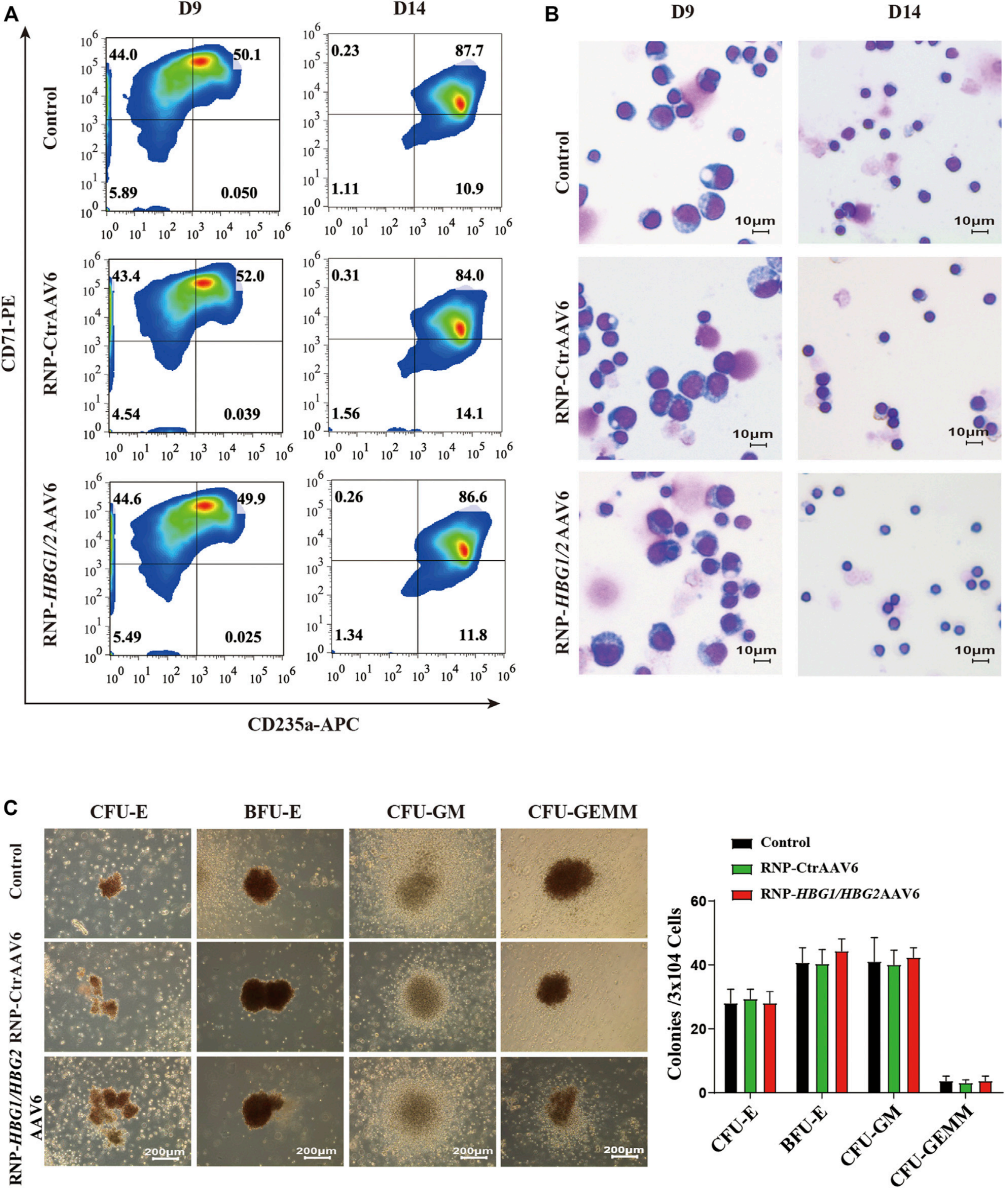
The impact of the genome editing strategy on the differentiation of CD34^+^ HSPCs. **(A)** Erythroid differentiation was measured on indicated days by flow cytometric analysis based on the expression of CD235a and CD71. The data reflect studies of CD34^+^ cells from β-thalassemia donors in three independent experiments, the representative plots are shown. **(B)** Representative images of Wright-Giemsa staining of different time points of differentiated CD34^+^ cells (objective lens, ×40). **(C)** Left: morphology of colonies at 14 days culture were observed at a magnification of ×40. Right: colonies were counted and classified according to their morphology (mean ± SD, *n* = 3).

We examined the effect of the genome editing strategy on erythroid differentiation. We observed that introducing HPFH mutations in the *HBG1/HBG2* promoters had no significant effect on erythrocyte maturation or terminal differentiation of CD34^+^ HSPCs derived from mobilized peripheral blood of two severe β-thalassemia patients by evaluating the markers CD235a and CD71 of erythroid lineage (Figure 3A). Furthermore, the morphology of erythrocytes after genome editing was comparable to wild-type cells (Figure 3B). Finally, We performed Colony Forming Cell (CFC) Assayto evaluate the effect of the genome editing strategy on the proliferation and differentiation pattern of HSPCs. We found that introducing HPFH mutations in the *HBG1/HBG2* promoters exerts no significant impact on frequency and proportion of colony forming unit (CFU)-erythroid (CFU-E) and burst forming unit-erythroid (BFU-E), CFU-granulocyte, macrophage (CFU-GM) and CFU-granulocyte, erythrocyte, macrophage, megakaryocyte (CFU-GEMM) in HSPCs cultured in a semisolid methylcellulose-based medium (Figure 3C). Taken together, these findings suggest that introducing HPFH mutations in the *HBG1/HBG2* promoters may be a promising therapeutic strategy to reactivate HbF without significantly altering erythrocyte differentiation.

## Discussion

Ongoing clinical gene therapy trial of autologous HSPCs genetically modified using lentiviral vectors expressing β-like globin transgenes are showing encouraging outcomes (Ribeil et al., 2017; Thompson et al., 2018). However, the potential risk of genotoxic complications caused by the insertional mutagenesis with randomly integrating viral vectors cannot be underestimated. Another Phase I clinical trials for sickle cell disease and β-thalassemia which reactive erythroid HbF expression by Cas9-mediated disruption of the HbF-repressor element BCL11A are now under way, and early data also shows promising results (Frangoul et al., 2021). However, the approaches are still in their infancy, and safety and effectiveness issues remain to be determined and must be addressed before being translated into clinical practice. Here, we achieved efficient gene editing (>80%) with no detectable genotoxicity in HSPCs by combining the safer tools AAV6 and RNP to mimic the beneficial naturally occurring HPFH to reactivate HbF instead of direct disruption the suppressors, which may provide a novel therapeutic option and advance the clinical translation of genome editing therapy for β-thalassemia.

It was previously reported that Cas9-mediated disruption of the repressor BCL11A in the *HBG1/HBG2* promoters significantly induced fetal hemoglobin expression. However, Cas9-mediated disruption of BCL11A binding sites is accompanied by various NHEJ-related InDels, some of which might not lead to a significant increase in γ-globin expression as indicated by the monoclonal experiment (Traxler et al., 2016). Based on the generation of double-strand break (DSB) by Cas9-RNP, we realized a greater extent of γ-globin reactivation by delivery of AAV6 homologous donor to integrate six specific natural HPFH mutations in the *HBG1* and *HBG2* promoters. As expected, the combination of *HBG1/HBG2* AAV6 and Cas9-RNP created HDR-related genotypes that further reactivate γ-globin, which may translate to greater clinical therapeutic benefits. In addition, Traxler et al., and Métais et al., demonstrated that high efficiency of disruption of the BCL11A binding site can be achieved in SCD CD34^+^ HSPCs (Traxler et al., 2016; Métais et al., 2019). However, the strategy has not been tested in β-thalassemia patient cells. In our study, high-efficient editing of HSPCs *in vitro* and post-transplantation reconstruction of hematopoietic system *in vivo* enriched preclinical data for gene therapy of β-thalassemia.

In summary, our studies demonstrate that introducing six specific HPFH mutations leads to significant re-activation of γ-globin and thus elevates HbF in both HUDEP-2 cells and HSPCs derived from β-thalassemia major patients. We found that the gene editing strategy does not lead to observable alteration in erythroid differentiation of CD34^+^ HSPCs. Moreover, *in vivo* studies indicated that the assays maintained long-term hematopoietic reconstitution in the edited cells. These findings may facilitate the clinical transformation of genome editing-based treatment of hemoglobinopathies.

## Data Availability

The original contributions presented in the study are included in the article/Supplementary Material, further inquiries can be directed to the corresponding authors.
