# Supplementary material for: Induction of Fetal Hemoglobin by Introducing Natural Hereditary Persistence of Fetal Hemoglobin Mutations in the γ-Globin Gene Promoters for Genome Editing Therapies for β-Thalassemia
Source: Front Genet. 2022 May 17;13:881937. doi: 10.3389/fgene.2022.881937 (PMC9152165; doi:10.3389/fgene.2022.881937)
Supplement: Supplementary file 1 [file Table1.DOCX]

**Supplemental Materials**

**Genome editing of HUDEP-2 or CD34**^+^ **HSPCs**

HSPCs and HUDEP-2 cells were cultured and differentiated into erythroid cells as previously described (Cheng et al. 2019). TrueCut Cas9 protein was purchased from Invitrogen. Chemically modified sgRNA, including 2'-O-methyl 3'-phosphorothioate modifications at both the 5' and 3' ends, was purchased from Synthego (Menlo Park, CA, USA). The target sequences for sgRNA were as follows: 5'-CCTTGACCAATAGCCTTGACAAG-3'. Ribonucleoprotein complexes were formed by incubating 12 pmol Cas9 protein with 64 pmol sgRNA in 5 μL Buffer T (Thermo Fisher Scientific) for 20 min. Approximately 2 × 10^5^ cells were resuspended in the T buffer and mixed with RNP electroporated with 1600 V, 3 pulses of 10 ms in a final volume of 10 μL using a Neon Transfection System. Following electroporation, AAV6 vectors (Vector Builder, Guangzhou, China) were immediately supplied to the cells at 1 × 10^4^ vector genomes per cell.

**Deep sequencing of on- and off-target sites**

Cells were harvested three to five days after targeting, and "Amplification Best Companion" (Mei5 Bioservices, Beijing, China) was used in DNA isolation. For the on-target sites, to avoiding amplification of AAV6 fragments, nested PCR was performed to amplify editing sites at *HBG1/2* promoters along with KOD One PCR Master Mix (TOYOBO, Osaka, Japan). The first pair of PCR primers were designed outside the homologous arms and amplify an approximately 4-kb fragment:

Forward: 5'-CCAATATGTCAGAAACAGCACTG -3'; and

Reverse: 5'-AAACCTGAGATAAACATGGT-3';

### The 4-kb fragment was [extracte](https://www.baidu.com/link?url=PVChAXX9PZYuVOUVt01dKpQUwVeoQ_FNjhgkI7aYdksrgYa1_3xWtAJe5-5vnmg5rZ8F0C4u5doFcy5b8jRCAK&wd=&eqid=c7f61aa2000632140000000661bc3251)d from the first PCR products by DNA Gel Extraction Kit (TIANGEN, Beijing, China). Next, the nested primers amplify a 200-bp fragment containing the edited sites:

Forward: 5'-GGAGTGAGTACGGTGTGCATCGGAACAAGGCAAAGGCTAT-3'; and

Reverse: 5'-GAGTTGGATGCTGGATGGCCTGGCCTCACTGGATACTCTA-3'. Chimeric mutation frequency was determined by NGS at a 10000× reads depth (Liu et al. 2019). For the off-target sites, NGS were analyzed for top-ranked off-target sites predicted on http://chopchop.cbu.uib.no/.

**Assessment of human engraftment.**

1. NDG hTHPO mice were purchased from The Biocytogen (Catalogue no. 110590, Beijing, China). Post-transplantation chimerism was evaluated at week 16 in the bone marrow harvested from mice femur after euthanasia. Human cell lineage was tested in the mice bone marrow by staining using human antibodies, CD45 (368506, BioLegend, San Diego, CA, USA), CD33 (303436, BioLegend), CD19 (152409, BioLegend), and CD235a (349114, BioLegend). hCD235a^+^ erythrocytes sorted from post-transplantation B-NDG hTHPO bone marrow were harvested to extract RNA for RT-qPCR analysis. hCD45^+^ cells were sorted for genotyping.

**RNA isolation and RT-qPCR**

RNA was extracted from harvested cells using TRIzol regent (Invitrogen, Carlsbad, CA, USA) following the manufacturer’s instructions. cDNA was synthesized using a PrimeScript RT Reagent Kit (Takara Bio, Tokyo, Japan). qPCR was performed using TB Green^TM^ Premix Ex Taq^TM^ (Tli RNaseH Plus) (Takara Bio) and a Viia7 Real-Time PCR system (Thermo Fisher Scientific). Expression of *GAPDH*, *HBG*, and *HBB* genes were quantified by qPCR using the following synthesized primers:

*HBG*-F: 5'-CTGACTTCCTTGGGAGATGC-3';

*HBG*-R: 5'-TCCCAGGAGCTTGAAGTTCTC-3';

*HBB*-F: 5'-TACATTTGCTTCTGACACAAC-3';

*HBB*-R: 5'-ACAGATCCCCAAAGGAC-3';

*GAPDH*-F: 5'-GTGAAGGTCGGAGTCAACG-3';

*GAPDH*-R: 5'-TGAGGTCAATGAAGGGGTC-3'

**Colony formation assay**

3×10^4^ cells were suspended in MethoCult™ Media (StemCell Technologies, Vancouver, Canada) three to five days after targeting, and plated in 35-mm culture dishes. The cells in dishes were incubated for 14 days at 37°C with 5% CO2 in air and 95% humidity. Colonies in the dishes were counted using microscope and classified as colony-forming unit-erythroid (CFU-E), burst-forming unit-erythroid (BFU-E), colony-forming unit-granulocyte macrophage (CFU-GM), and colony-forming unit-granulocyte erythroid macrophage megakaryocyte (CFU-GEMM) according to their morphology.

**Giemsa stain**

Thin films of differentiated HSPCs were prepared and air-dried on glass slides for 2-3 min. The cells were then incubated with 60μl Wright-Giemsa Solution A (BASO, Zhuhai, China) for 50s. And then, Wright-Giemsa Solution B (3-4 times of Solution A) is added onto Solution A and mix [thoroughly](https://www.powerthesaurus.org/thoroughly/synonyms) to stain for 5-10 min. Slides were rinsed with tap water and then photographed under a microscope (Leica Microsystems, Switzerland).

**Flow cytometry analysis**

After washing with PBS, differentiated cells derived from CD34^+^ HSPCs were incubated with the APC-conjugated anti-CD235a antibody (BioLegend, San Diego, CA, USA), PE-conjugated anti-CD71 antibody (BD Biosciences, New Jersey, USA) for 30 min at RT. Cells were washed for twice following incubation and measured by the Invitrogen^TM^ Attune^TM^ NxT flow cytometry system.

**The core homologous recombination sequences of *HBG1* AAV6 and *HBG2* AAV6 donors.**

Within the sequences of *HBG1* AAV6 and *HBG2* AAV6 donors, the homology arms sequences are grey, the six specific HPFH-associated mutations are yellow.

*HBG1* AAV6 donor:

GTGTGGACTATTAGTCAATAAAAACAACCCTTGCCTCTTTAGAGTTGTTTTCCATGTACACGCACATCTTATGTCTTAGAGTAAGATTCCCTGAGAAGTGAACCTAGCATTTATACAAGATAATTAATTCTAATCCACAGTACCTGCCAAAGAACATTCTACCATCATCTTTACTGAGCATAGAAGAGCTACGCCAAAACCCTGGGTCATCAGCCAGCACACACACTTATCCAGTGGTAAATACACATCATCTGGTGTATACATACATACCTGAATATGGAATCAAATATTTTTCTAAGATGAAACAGTCATGATTTATTTCAAATAGGTACGGATAAGTAGATATTGAGGTAAGCATTAGGTCTTATATTATGTAACACTAATCTATTACTGCGCTGAAACTGTGGCTTTATAGAAATTGTTTTCACTGCACTATTGAGAAATTAAGAGATAATGGCAAAAGTCACAAAGAGTATATTCAAAAAGAAGTATAGCACTTTTTCCTTAGAAACCACTGCTAACTGAAAGAGACTAAGATTTGTCCCGTCAAAAATCCTGGACCTATGCCTAAAACACATTTCACAATCCCTGAACTTTTCAAAAATTGGTACATGCTTTAGCTTTAAACTACAGGCCTCACTGGAGCTAGAGACAAGAAGGTAAAAAACGGCTGACAAAAGAAGTCCTGGTATCCTCTATGATGGGAGAAGGAAACTAGCTAAAGGGAAGAATAAATTAGAGAAAAACTGGAATGACTGAATCGGAACAAGGCAAAGGCTATAAAAAAAATTAGCAGTATCCTCTTGGGGGCCCCTCCCGCACACTATCTCAATGCAAACATCTGTCTGAAACGGTCCCTGGCTAAACTCCACCCATGGGTTGGCCAGCCTTGCCTTAACTGATAGCCTTGACAAGGCAAACTTGACCAATAGTCTTAGAGTATCCAGTGAGGCCAGGGGCCGGCGGCTGGCTAGGGATGAAGAATAAAAGGAAGCACCCTTCAGCAGTTCCACACACTCGCTTCTGGAACGTCTGAGGTTATCAATAAGCTCCTAGTCCAGACGCCATGGGTCATTTCACAGAGGAGGACAAGGCTACTATCACAAGCCTGTGGGGCAAGGTGAATGTGGAAGATGCTGGAGGAGAAACCCTGGGAAGGTAGGCTCTGGTGACCAGGACAAGGGAGGGAAGGAAGGACCCTGTGCCTGGCAAAAGTCCAGGTCGCTTCTCAGGATTTGTGGCACCTTCTGACTGTCAAACTGTTCTTGTCAATCTCACAGGCTCCTGGTTGTCTACCCATGGACCCAGAGGTTCTTTGACAGCTTTGGCAACCTGTCCTCTGCCTCTGCCATCATGGGCAACCCCAAAGTCAAGGCACATGGCAAGAAGGTGCTGACTTCCTTGGGAGATGCCACAAAGCACCTGGATGATCTCAAGGGCACCTTTGCCCAGCTGAGTGAACTGCACTGTGACAAGCTGCATGTGGATCCTGAGAACTTCAAGGTGAGTCCAGGAGATGTTTCAGCCCTGTTGCCTTTAGTCTCGAGGCAACTTAGACAACGGAGTATTGATCTGAGCACAGCAGGGTGTGAGCTGTTTGAAGATACTGGGGTTGGGGGTGAAGAAACTGCAGAGGACTAACTGGGCTGAGACCCAGTGGTAATGTTTTAGGGCCTAAGGAGTGCCTCTAAAAATCTAGATGGACAATTTT

*HBG2* AAV6 donor:

CACAGTGTGTGGACTATTAGTCAATAAAACAGTCCCTGCCTCTTAAGAGTTGTTTTCCATGCAAATACATGTCTTATGTCTTAGAATAAGATTCCCTAAGAAGTGAACCTAGCATTTATACAAGATAATTAATTCTAATCCATAGTATCTGGTAAAGAGCATTCTACCATCATCTTTACCGAGCATAGAAGAGCTACACCAAAACCCTGGGTCATCAGCCAGCACATACACTTATCCAGTGATAAATACACATCATCGGGTGCCTACATACATACCTGAATATAAAAAAAATACTTTTGCTGAGATGAAACAGGCGTGATTTATTTCAAATAGGTACGGATAAGTAGATATTGAAGTAAGGATTCAGTCTTATATTATATTACATAACATTAATCTATTCCTGCACTGAAACTGTTGCTTTATAGGATTTTTCACTACACTAATGAGAACTTAAGAGATAATGGCCTAAAACCACAGAGAGTATATTCAAAGATAAGTATAGCACTTCTTATTTGGAAACCAATGCTTACTAAATGAGACTAAGACGTGTCCCATCAAAAATCCTGGACCTATGCCTAAAACACATTTCACAATCCCTGAACTTTTCAAAAATTGGTACATGCTTTAACTTTAAACTACAGGCCTCACTGGAGCTACAGACAAGAAGGTGAAAAACGGCTGACAAAAGAAGTCCTGGTATCTTCTATGGTGGGAGAAGAAAACTAGCTAAAGGGAAGAATAAATTAGAGAAAAATTGGAATGACTGAATCGGAACAAGGCAAAGGCTATAAAAAAAATTAAGCAGCAGTATCCTCTTGGGGGCCCCTCCCGCACACTATCTCAATGCAAACATCTGTCTGAAACGGTCCCTGGCTAAACTCCACCCATGGGTTGGCCAGCCTTGCCTTAACTGATAGCCTTGACAAGGCAAACTTGACCAATAGTCTTAGAGTATCCAGTGAGGCCAGGGGCCGGCGGCTGGCTAGGGATGAAGAATAAAAGGAAGCACCCTTCAGCAGTTCCACACACTCGCTTCTGGAACGTCTGAGGTTATCAATAAGCTCCTAGTCCAGACGCCATGGGTCATTTCACAGAGGAGGACAAGGCTACTATCACAAGCCTGTGGGGCAAGGTGAATGTGGAAGATGCTGGAGGAGAAACCCTGGGAAGGTAGGCTCTGGTGACCAGGACAAGGGAGGGAAGGAAGGACCCTGTGCCTGGCAAAAGTCCAGGTCGCTTCTCAGGATTTGTGGCACCTTCTGACTGTCAAACTGTTCTTGTCAATCTCACAGGCTCCTGGTTGTCTACCCATGGACCCAGAGGTTCTTTGACAGCTTTGGCAACCTGTCCTCTGCCTCTGCCATCATGGGCAACCCCAAAGTCAAGGCACATGGCAAGAAGGTGCTGACTTCCTTGGGAGATGCCATAAAGCACCTGGATGATCTCAAGGGCACCTTTGCCCAGCTGAGTGAACTGCACTGTGACAAGCTGCATGTGGATCCTGAGAACTTCAAGGTGAGTCCAGGAGATGTTTCAGCACTGTTGCCTTTAGTCTCGAGGCAACTTAGACAACTGAGTATTGATCTGAGCACAGCAGGGTGTGAGCTGTTTGAAGATACTGGGGTTGGGAGTGAAGAAACTGCAGAGGACTAACTGGGCTGAGACCCAGTGGCAATGTTTTAGGGCCTAAGGAGTGCCTCTGAAAATCTAGATGGACAACTTT

**Reference:**

Cheng Y, Shang X, Chen D, Pang D, Zhao C, Xu X. MicroRNA-2355-5p regulates γ-globin expression in human erythroid cells by inhibiting KLF6. *Br J Haematol*. 2020;193(2):401-405.

Liu Q, Wang C, Jiao X, Zhang H, Song L, Li Y, et al. Hi-TOM: a platform for high-throughput tracking of mutations induced by CRISPR/Cas systems. *Sci China Life Sci*. 2019;62(1):1-7.
